# Down-regulation of granulocyte-macrophage colony-stimulating factor by 3C-like proteinase in transfected A549 human lung carcinoma cells

**DOI:** 10.1186/1471-2172-12-16

**Published:** 2011-02-17

**Authors:** Hsien-Hua Liao, Yao-Chen Wang, Miles Chih-Ming Chen, Hsien-Yu Tsai, Johnson Lin, Shui-Tein Chen, Gregory Jiazer Tsay, Sun-Long Cheng

**Affiliations:** 1 Institute of Medicine, Chung Shan Medical University, Taichung 40242, Taiwan; 2 Department of Surgery, Chung Shan Medical University Hospital, Taichung 40242, Taiwan; 3 School of Medicine, Chung Shan Medical University, Taichung 40242, Taiwan; 4 Department of Internal Medicine, Chung Shan Medical University Hospital, Taichung 40242, Taiwan; 5 Institute of Biological Chemistry, Academia Sinica, Taipei, 11529, Taiwan; 6 Institute of Biochemical Sciences, College of Life Science, National Taiwan University, Taipei, 10617, Taiwan; 7 School of Biochemistry, Genetics, Microbiology and Plant Pathology, University of KwaZulu-Natal (Westville Campus), Private Bag X54001, Durban, Republic of South Africa; 8 Genomics Research Center, Academia Sinica, Taipei, 11529, Taiwan; 9 Institute of Immunology, Chung Shan Medical University, Taichung, 40242, Taiwan; 10 Department of Plastic Surgery, Chung Shan Medical University Hospital, Taichung, 40242, Taiwan

## Abstract

**Background:**

Severe Acute Respiratory Syndrome (SARS) is a severe respiratory illness caused by a novel virus, the SARS coronavirus (SARS-CoV). 3C-like protease (3CL^pro^) of SARS-CoV plays a role in processing viral polypeptide precursors and is responsible of viral maturation. However, the function of 3CL^pro ^in host cells remains unknown. This study investigated how the 3CL^pro ^affected the secretion of cytokines in the gene-transfected cells.

**Results:**

From immunofluorescence microscopy, the localization of c-myc tagged 3CL^pro ^was detected both in the cytoplasm and nucleus of transfected A549 cells. Expression of granulocyte-macrophage colony-stimulating factor (GM-CSF) was significantly decreased in 3CL^pro^-transfected cells by both RT-PCR and ELISA, but without changes in other cytokines, *i.e*., IL-1β, IL-6, IL-8, IL12p40, TNF-α, and TGF-β. Furthermore, the protein levels of NF-kB decreased in 3CL^pro^-transfected A549 cells when compared to EGFP transfected cells.

**Conclusions:**

Our results suggest that the 3CL^pro ^may suppress expression of GM-CSF in transfected A549 cells through down-regulation of NF-kB production.

## Background

Severe acute respiratory syndrome (SARS) emerged as a communicable human disease in November 2002 and rapidly spread throughout the world [1]. A coronavirus, called SARS-associated coronavirus (SARS-CoV), was identified as the causative agent [2,3]. SARS-CoV is a plus-strand RNA virus featuring a large single-stranded RNA genome of approximately 29.7 kb [4] that contains 16 non-structural proteins (nsps) with multiple enzymatic functions. These are known or are predicted to include types of enzymes that are common components of the replication machinery of plus-strand RNA viruses [5,6]. In these nsps, the Nsp5 [main protease (M^pro^) or 3C-like protease (3CL^pro^)] is responsible for the proteolytic processing of viral polypeptides into functional proteins and 3CL^pro ^mutant, C145A, blocks the maturation process [7]. This viral protease has been the target for design of the inhibitors [7-11]. Recently, SARS-3CL^pro ^was found to induce cell apotosis and interact with cellular vacuolar-H^+ ^ATPase [12,13]. Several human proteins might possess a similar structure of SARS-3CL^pro ^cleavage sites using computational methods [14]. Theses studies implied the SARS-3CL^pro ^could involve in virus-induced host pathology.

SARS-CoV causes a severe lung infection, and the epithelial cells of the upper respiratory tract are the primary targets [15-17]. SARS-CoV reproduces rapidly and causes massive cell damage. At the whole-body level, immune-mediated damage, due to activation of cytokines and/or chemokines [18-22] and, perhaps, autoimmunity [23,24], may play key roles in the clinical and pathological features of SARS. One of the major causes of host death is the induction of lung fibrosis. The mechanism of the lung fibrosis caused by SARS-CoV requires elucidation. Significant efforts have been made to analyze the function of SARS-3CL^pro ^on viral replication, but the impact of SARS-3CL^pro ^on cytokines of the host cell is still unclear. In this study, we constructed the pEGFP-C3 vector with SARS-3CL^pro ^gene and transfected it into A549 lung epithelial cells. The expression profiles of cytokines were validated by RT-PCR and ELISA. The results indicated that the expression and secretion of granulocyte-macrophage colony-stimulating factor (GM-CSF) decrease in SARS-3CL^pro ^transfected A549 cells. Furthermore, NF-kB, putative regulators of GM-CSF gene expression, was also decreased on its protein level in 3CL^pro ^transfected cells.

## Results

### Construction of SARS-CoV 3CL^pro ^wild type and a mutant C145A clone into pEGFP-C3 vector

**Figure 1:**
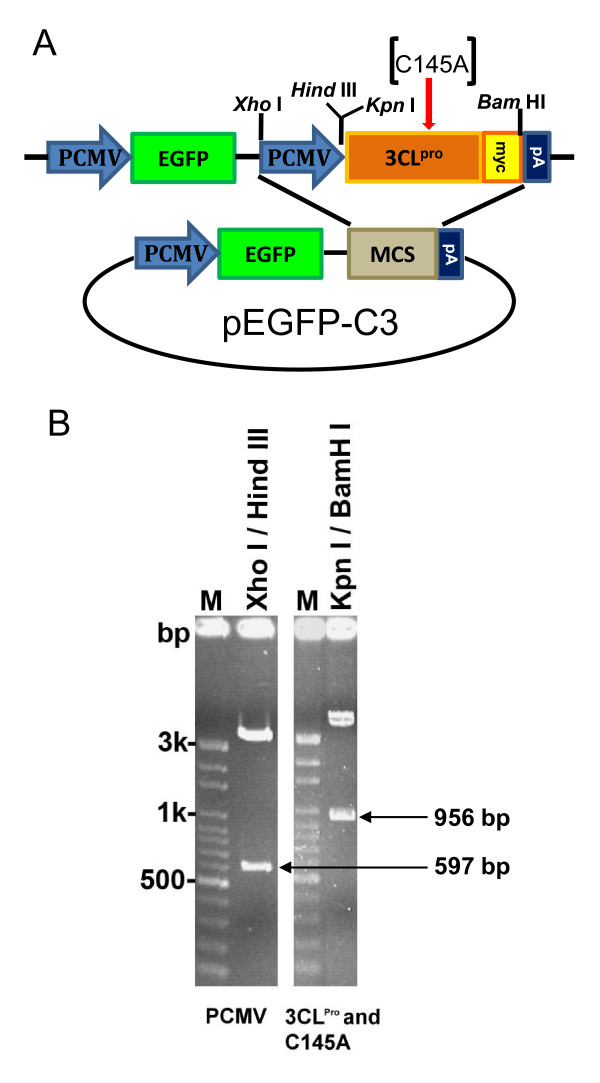
**Construction of pEGFP-3CL^pro ^and pEGFP-C145A expression vectors**. (A) Scheme of pEGFP-3CL^pro ^and pEGFP-C145A construction. PCMV, human cytomegalovirus (CMV) immediate early promoter; EGFP, enhanced green fluorescent protein gene; MCS, multiple cloning sites; 3CL^pro^, SARS-CoV 3CL^pro ^gene; C145A, 3CL^pro ^C145A mutant gene; myc, myc-tag sequence; pA, SV40 early mRNA polyadenylation signal sequence. (B)The constructs were digested with restriction enzyme and separated on 1% agarose gel. M, DNA markers.

To optimize the efficiency of transfection and expression of functional 3CL^pro ^in A549 lung cancer cell line, we constructed expression vector expressing SARS-CoV 3CL^pro ^wild type gene into a pEGFP-C3 vector (Figure 1A). An inactive 3CL^pro ^mutant, C145A (TGT→GCT), was also included in this study as a negative control [7]. Furthermore, the myc tag sequence was also introduced into the 3' end of the 3CL^pro ^and C145A genes for immunofluorescence analysis. The DNA fragments of CMV promoter, 3CL^pro ^wild type, and C145A 3CL^pro ^mutant were amplified by PCR using primers containing restriction enzyme sites as shown in Figure 1A. The expression constructs were further verified by 1% agarose gel electrophoresis after restriction enzymes digestion (Figure 1B). As expected, the 597 bp of PCMV and 956 bp of 3CL^pro ^(and C145A) fragments were excised from plasmids with appropriate restriction enzymes and were confirmed by DNA sequencing.

### Expression of EGFP, 3CL^pro^, and C145A in A549 cells

**Figure 2:**
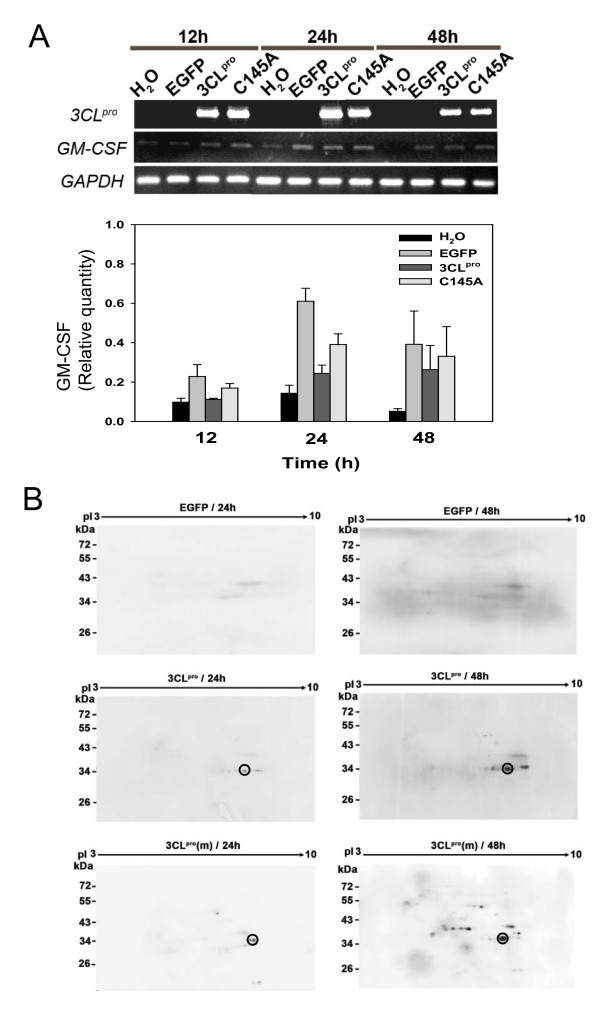
**RT-PCR and 2-D western blot analysis of EGFP, 3CL^pro^, and C145A transfected A549 cell line**. (A) RT-PCR analysis of 3CL^pro ^and GM-CSF genes expression at 12, 24, and 48 hrs in A549 cells which were transfected with EGFP, 3CL^pro^, and C145A respectively. The relative abundance compared with the expression level of GAPDH is depicted in the bar chart below. (B) 2-D western blots of 3CL^pro ^and C145A expression patterns in A549 cells. A549 cells transfected with EGFP, 3CL^pro ^and C145A were analyzed at 24 and 48 hrs after transfection. Proteins were resolved by 2-D gels and electroblotted onto PVDF membranes. Western blots were probed with anti-3CL^pro ^specific antibody. The circle indicates the site of 3CL^pro^.

**Figure 3:**
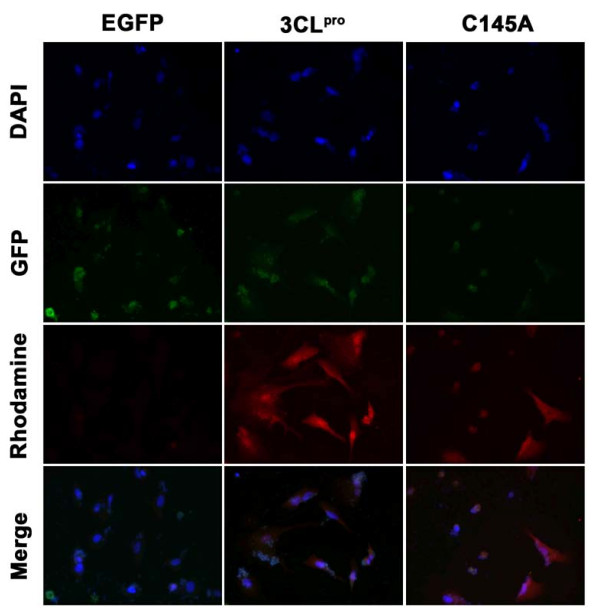
**Nuclear and cytoplasmic localization of 3CL^pro ^and C145A in transfected A549 cells**. EGFP, 3CL^pro^, and C145A transfected A549 cells were collected at 24 hrs after transfection. Cells were fixed and stained as described in Methods. Nuclei were stained with DAPI (blue fluorescence, obtained from the excitation of 359 nm and emission of 468 nm.). GFP panel shows the green fluorescence obtained from the excitation of 488 nm and emission of 509 nm. 3CL^pro ^and C145A were detected with a rhodamine-conjugated secondary antibody (red fluorescence, obtained from the excitation of 496 nm and emission of 520 nm).

The plasmids of pEGFP-C3, pEGFP-3CL^pro^, and pEGFP-C145A were successfully transfected into A549 cells respectively. The expression levels of 3CL^pro ^and C145A mRNA were determined by reverse transcription (RT)-PCR and the protein expression levels were analyzed using 2D western blots (Figure 2A and 2B). The results indicated that the expression of 3CL^pro ^and C145A mRNA reached the highest level in A549 cells at 12 to 24 hrs and slightly decreased their expression at 48 h after transfection. However, 2D western blots results indicated that the protein expression levels of 3CL^pro ^and C145A steadily increased with time up to 48 hrs after transfection. To track the locations of 3CL^pro ^and C145A proteins in transfected A549 cells, Myc-tagged 3CL^pro ^and C145A were detected using rhodamine-conjugated anti-Myc antibodies. The immunofluorescence microscopic images demonstrated 60-70% transfection efficiency (data not shown) and the localizations of c-myc tagged 3CL^pro ^and C145A were detected both in the cytoplasm and nucleus of transfected A549 cells compared to control EGFP (Figure 3).

### The expression of GM-CSF in SARS-3CL^pro ^transfected A549 cells by semi-quantification RT-MPCR

**Figure 4:**
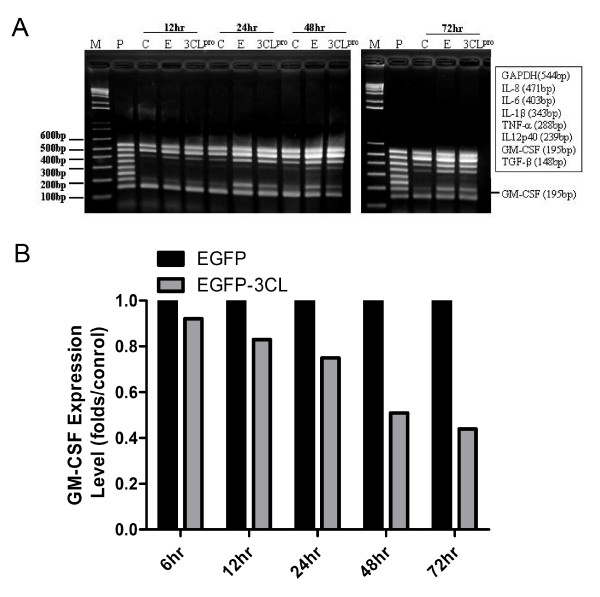
**Expression of various cytokines and chemokines in 3CL^pro ^transfected A549 cells using RT-PCR**. (A) Total RNAs were harvested at 12, 24, 48 and 72 hrs. RT-PCR was performed to detect the expression of various cytokines and chemokines including IL-1β (343 bp), IL-6 (403 bp), IL-8 (471 bp), IL12p40 (239 bp), TNF-α (288 bp), TGF-β (148 bp), GM-CSF (195 bp). GAPDH was used as control. A549 cells were used as the background. (B) Expression of GM-CSF in 3CL^pro ^transfected A549 cells using RT-PCR. Total RNA was harvested at 6, 12, 24, 48 and 72 hrs. RT-PCR was performed to detect the mRNA expression of GM-CSF (277 bp). GAPDH was used to normalize the cell number (307 bp). There were marked decreases of GM-CSF in 3CL^pro ^transfected A549 cells after 24 hrs (*; p < 0.05). A549 cells were used as the background. C, A549 cells; E, EGFP transfected cells; 3CL^pro^, 3CL^pro ^transfected cells.

Total RNA from A549 cells (C), EGFP (E) and pEGFP-3CL^pro ^(3CL^pro^) transfected cells were collected at 12, 24, 48, and 72 hrs. As shown in Figure 4, expression of GM-CSF was demonstrated in 3CL^pro ^transfected cells, A549 cells and EGFP transfected cells (Figures 2A and 4A) by Multiplex PCR kits. The decreased percentage was observed in EGFP-3CL^pro ^transfected A549 cells compared to the EGFP transfected A549 cells at 6, 12, 24, 48, and 72 hrs is 9%, 17%, 25%, 49%, and 56%, respectively. There was significant decrease of GM-CSF in 3CL^pro ^transfected A549 cells after 24 hrs (p < 0.005). Additionally, there were no change to the mRNA expression of IL-1β, IL-6, IL-8, IL12p40, TNF-α, and TGF-β found in 3CL^pro ^transfected cells. To confirm the mRNA expression of GM-CSF, other primer pairs were used to amplify GM-CSF by semi-quantification RT-MPCR. As shown in Figure 4B, decreased expression of GM-CSF was noted in 3CL^pro ^transfected cells compared to GAPDH in A549 cells and EGFP transfected cells at 24, 48, and 72 hrs. A significant decrease of GM-CSF was observed in 3CL^pro ^transfected A549 cells after 24, 48, and 72 hrs with maximal reduction folds of vector control about 0.75, 0.53 and 0.45, respectively (*p *< 0.005).

### The secretion of GM-CSF in SARS-3CL^pro ^transfected A549 cells by ELISA

**Figure 5:**
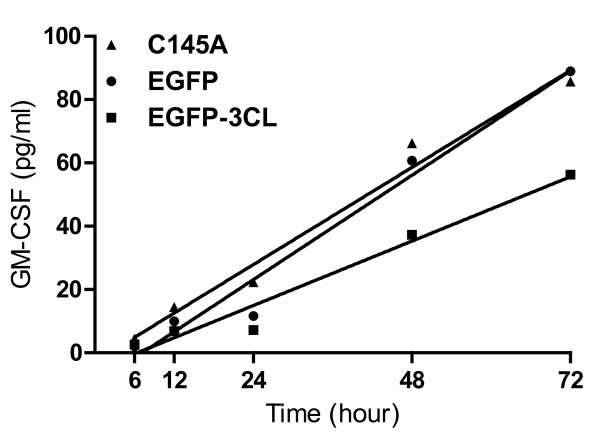
**Decreased secretion of GM-CSF in SARS-CoV-3CL^pro ^transfected cells**. A549 cells were transfected with EGFP or 3CL^pro ^or C145A plasmids. The supernatants were collected from cell cultures at 6, 12, 24, 48 and 72 hrs and ELISA was performed to evaluate the amount of secreted GM-CSF. The supernatant from the cell culture of A549 cells was used as a control. A significant decrease of GM-CSF was observed in 3CL^pro ^or C145A transfected A549 cells (p = 0.0204 and 0.0236 respectively).

As shown in Figure 5, the values of GM-CSF concentration in the culture mediums of EGFP transfected A549 cells at 6, 12, 24, 48, and 72 hrs were 2.047 ± 0.2(pg/ml), 9.928 ± 0.13(pg/ml), 11.657 ±.15 (pg/ml), 60.714 ±.0.18 (pg/ml), 88.926 ±.0.31 (pg/ml), respectively. The values of GM-CSF concentration in the culture mediums of 3CL^pro ^transfected A549 cells at 6, 12, 24, 48, and 72 hrs were 2.624 ± 0.13(pg/ml), 6.852 ± 0.17(pg/ml), 7.237 ±.0.11 (pg/ml), 37.313 ±.0.18 (pg/ml), 56.25 ±.0.31 (pg/ml), respectively. The values of GM-CSF concentration in the culture mediums of C145A transfected A549 cells at 6, 12, 24, 48, and 72 hrs were 4.431 ± 0.11(pg/ml), 14.424 ± 0.21(pg/ml), 22.281 ±.0.54 (pg/ml), 66.235 ±.0.68 (pg/ml), 85.715 ±.0.43 (pg/ml), respectively. A significant decrease of GM-CSF was observed in 3CL^pro ^transfected A549 cells compared to the EGFP-transfected and C145A-transfected A549 cells by an analysis of covariance (*p *= 0.0204 and 0.0236 respectively).

### Involvement of NF-κB in 3CL^pro^-mediated GM-CSF expression in A549 cells

**Figure 6:**
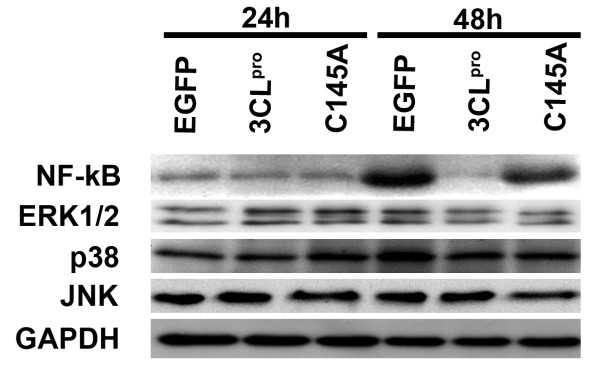
**Expression of NF-kB and MAPKs in EGFP, 3CL^pro^, and C145A tranfected A549 cells**. A549 cells tranfected with EGFP, 3CL^pro^, and C145A were harvested at 24 and 48 hrs as indicated above and total cell lysate were resolved in 12.5% SDS-PAGE. Antibodies used in western blotting are indicated in left panel.

To investigate whether ERK1/2, p38, and JNK MAPK pathways and NF-κB were involved in 3CL^pro^-mediated GM-CSF production in A549 cells [25,26], we examined the expression levels of these MAP kinases and NF-κB by western blot analysis. As shown in Figure 6 (a bar graph including mean and standard deviation were provided in Additional File 1), the expression of NF-κB was reduced in 3CL^pro ^transfected cells at 48 hrs after transfection compared to cells transfected with EGFP. In contrast, over expression of 3CL^pro ^did not change the expression levels of ERK1/2, p38, and JNK in A549 cells significantly.

## Discussion

Severe acute respiratory syndrome coronavirus (SARS-CoV) causes a severe lung infection [18-20]. It is important to elucidate the mechanism of the lung fibrosis caused by SARS-CoV. The expression of 3CL^pro ^and C145A in A549 cells was established (Figures 2 and 3) to elucidate the pathogenesis of SARS-CoV in lung epithelial cells. The localization patterns of 3CL^pro ^and C145A were observed both in the cytoplasm and nucleus of A549 cells by immunofluorescence (Figure 3). Decreased expression and levels of GM-CSF were found in 3CL^pro^-transfected cells using RT-PCR, but not in C145A transfected cells (Figure 2A). Down-regulation of GM-CSF expression by 3CL^pro ^in A549 cells may be attributed in part to the reduced protein level of NF-kB (Figure 6). In addition, there were no significant decreases in the mRNA expression of IL-1β, IL-6, IL-8, IL12p40, TNF-α, TGF-β using RT-MPCR. This finding is consistent with that of Ziegler *et al *[27] who reported no induction of cytokines genes (IFN-α, IFN-β, IL-28, IL-29, TNF-α, CCLl5 or CXCL10) or IFN-α/β-induced MxA gene in SARS-CoV-infected A549 cells.

GM-CSF was originally defined by its ability to generate *in vitro *granulocyte and macrophage colonies from bone marrow precursor cells [28]. Apart from its physiological role in the control of alveolar macrophage development, it now appears more likely that its major role lies in its ability to govern the properties of the more mature myeloid cells of the granulocyte and macrophage lineages, particularly during host defense and inflammatory reactions. Recent evidence for the key role of GM-CSF in inflammatory and autoimmune diseases is summarized [29]. GM-CSF-deficient mice showed an increased susceptibility to pulmonary infections [30,31]. In addition, some studies also demonstrated that impaired production of GM-CSF played a role in the development of bleomycin-induced pulmonary fibrosis [32,33]. Together, there is growing evidence suggesting that GM-CSF is an important regulator of the alveolar epithelium, surfactant homeostasis, and lung host defense [31,34]. To support the above, our experimental results show decreased expression and secretion of GM-CSF in 3CL^pro^-transfected cells, and suggest that SARS-3CL^pro ^will affect host defense.

Due to the functional importance of SARS-CoV 3CL^pro ^in proteolytic recognition and processing of the virus polyproteins, the experimental approaches and computational prediction of substrate cleavage sites of 3CL^pro ^become attractive topics in anti-SARS drug design [8-11,15,35]. It is clear that the 3CL^pro ^cleavage sites are consistent preferentially with relative position of P1'-Ser/Ala P1-Gln P2-Leu [35,36]. In this study, 3CL^pro ^transfected A549 cells decreased the protein levels of NF-kB with subsequent decline in GM-CSF mRNA and protein expression (Figures 4-6). The amino acid sequence analyses of NF-kB indicated that non-canonical 3CL^pro ^cleavage sites (such as P1'-Tyr/Gln/Arg/Ile P1-Gln P2-Leu) commonly existed in NF-kB. Interestingly, NF-kB are associated in GM-CSF production in lung cancer cell lines [37]. In depth investigations are required to determine whether 3CL^pro ^directly or indirectly affects the protein levels of NF-kB in transfected A549 cell.

## Conclusions

The role of the viral cytopathic effect in the pathogenesis of SARS-CoV-associated lung diseases involvement remains unclear. Our results reveals that the 3CL^pro ^-induced decreased GM-CSF herein may provide further information on the pathogenesis of lung diseases with associated SARS-CoV. Also, the protein levels of NF-kB decreased in 3CL^pro ^transfected cells. Thus, we conclude that 3CL^pro ^may mediate down-regulation of NF-kB production and subsequently suppress GM-CSF mRNA and protein expression in transfected A549 cells. Therefore, the SARS-CoV 3CL^pro ^may play a role in the mechanism of lung fibrosis of SARS through the suppression of GM-CSF.

## Methods

### Plasmids

Plasmid pEGFP-C3 was obtained from CLONTECH (CLONTECH Laboratories, Palo Alto, CA, USA). This plasmid contains the EGFP variant and neomycin resistant genes under the control of the cytomegalovirus early gene promoter and the SV40 early gene promoter, respectively. Full-length 3CL^pro ^versions (wild type and mutant, C145A) and CMV promoter were amplified by PCR using primers containing suitable restriction sites for subcloning into the pEGFP-C3 vector (3CL^pro ^primers: 5' ATATGGTACCAgTGGTTTTAGGAAAATGGCATTC 3'/Kpn I, 5' GAGATGAGTTTCTGCTCTTGGAAGGTAACACCAGAGCATT 3', and ATATGGATCCCAGATCCTCTTCTGAGATGAGTTTCTGCTCTTGGAA 3'/BamH I; CMV promoter primers: 5' ATATCTCGAGTAGTTATTAATAGTAATCAATTAC 3'/XhoI and 5' ATATAAGCTTTAGCGCTAGCGGATCTGA 3'/Hind III). The PCR was performed with reagents containing a 0.2 μM primers mixture, 1.25 μM dNTP mixture, 1.5 μM MgCl**_2_**, 10 ng templates, and 2.5 U DNA polymerase (Takara, Tokyo, Japan). Amplified DNA fragments were then ligated into the cloning site of the pEGFP-C3 vector (EGFP) and transformed into *Escherichia coli *DH5α competent cells, which were obtained from Life Technologies (Carlsbad, California, USA). Restriction enzyme digestion, PCR and DNA sequencing analysis (Mission Biotech, Taipei, Taiwan) were used to verify the plasmids.

### Cell culture and Transfection

The human lung carcinoma A549 cells were obtained from American type culture collection (ATCC) (Manassas, VA, USA) and were grown in Dulbecco's modified Eagle medium (DMEM) supplemented with 10% fetal bovine serum (FBS) (GIBCO-BRL, Carlsbad, California, USA) at 37°C in a 5% CO_2 _incubator. A total of 1 × 10^6 ^cells were grown to 70% confluence in 100 mm^2 ^culture plates before transfection. The transfection reaction was performed by using Lipofectamine plus reagents (Invitrogen, California, USA) with 2 μg of each plasmid, pEGFP-C3, pEGFP-3CL^pro^, and pEGFP-C145A according to the manufacturer's instructions. The cells were then cultured in serum-free DMEM for 12 hrs at 37°C in a 5% CO_2 _incubator and subsequently in DMEM with 10% FBS.

### Immunofluorescence cell staining and Fluorescence Microscopy

EGFP, 3CL^pro ^and C145A transfected A549 cells were cultured on sterile cover slides for overnight and fixed by methanol. Samples were blocked with 10% blocking serum and incubated with 3CL^pro ^antibody (Abcam Inc., Cambridge, MA) and rhodamine conjugated second antibody (Invitrogen Corporation; Grand Island, NY) separately. Nuclei were stained with DAPI (4',6-diamidino-2-phenylindole, Sigma, St. Louis, MO). All transfected A549 cells were observed with a Zeiss Axioplan-2 epifluorescence microscope equipped with appropriate fluorescence filters. Digital images of the cells were recorded by using a spot camera system.

### mRNA analysis and RT-MPCR

All studies were carried out in a designated PCR-clean area. After treatments at 12-72 hrs, cell pellets were washed in DPBS twice and treated with Trizol mRNA lysis buffer for total RNA extraction. All samples were analyzed by reverse transcriptase Multiplex PCR (RT-MPCR) with messageScreenTM Human Inflammatory Cytokine Set 1 Multiplex PCR kits (Biosource, International, Inc., CA, USA) for a temporal and spatial distribution of mRNA expression of different cytokines. This method is an accurate and valid system to detect multiple gene expression by amplifying all the genes under the same conditions. The PCR derived DNA fragments, obtained by 25 PCR cycles, and was subjected to electrophoresis in a 1.7% agarose gel. The cDNAs encoding human granulocyte-macrophage-colony-stimulating factor (GM-CSF) and GAPDH were amplified by RT-PCR using the following primer pairs: 5'-TGAGTAGAGACACTGCTGCTG-3' (forward primer for GM-CSF cDNAs), 5'-TCAAAGGGGATGACAAGCAGAA-3' (reverse primer for GM-CSF cDNAs), 5'-CATGTTCGTCATGGGTGTGA-3' (forward primer for GAPDH cDNAs), and 5'-AGTGAGCTTCCCGTTCAGCTC-3' (reverse primer for GAPDH cDNAs). The amplification was performed in a 50 μl reaction volume containing 1× reaction buffer (Promega, Madison, Wisconsin, USA), 1.5 μM of MgCl_2_, 200 μM of dNTPs, 1 μM of each primer and 2.5 units of Taq DNA polymerase (Promega, Madison, Wisconsin, USA) using a Perkin-Elmer Gene Amp PCR system 2400. Each cycle consists of denaturation at 95°C for 1 min, annealing at 55°C for 45 sec, and amplification at 72°C for 45 sec. The RT-PCR derived DNA fragments obtained by 30 PCR cycles were subjected to electrophoresis on a 1.7% agarose gel. Following staining with ethidium bromide, the gels were photographed and band intensity was measured under UV light using the Alphaimager 2200 (Alphalnnotech, San Leandro, CA, USA). The specific RNA level of every sample was expressed as the product's intensity. cDNA encoding glyceraldehyde- 3-phosphate dehydrogenase (GAPDH) was quantified and used to normalize the expression of GA-CSF and each cytokine for each sample.

### Cytokine ELISA

The quantification of cytokine levels from the cell culture mediums was performed by duplication using ELISA kits for GM-CSF (Biosource International, Inc, CA, USA), according to the manufacturer's instructions. In each case, the optical density of known standards was used to construct a calibration curve and the mean cytokine values ± SD were then calculated for each sample.

### Statistical analysis

Statistical analysis was performed using the paired t-test. A P value less than 0.05 was considered significant. GraphPad Prism software v5.01 (GraphPad Software Inc., USA) was employed for the analysis of covariance by comparing linear regression lines. Because the slopes differ so much, it is not possible to test whether the intercepts differ significantly.

### 2-DE

The 2-DE procedure was performed as described elsewhere with minor modification [38]. Briefly, 100 μg of protein was loaded onto IPG strips (18 cm, pH 3-10, GE Healthcare) for analytic 2-DE analysis. The IPG strips were subsequently rehydrated on the IPGphor IEF system (GE Healthcare, Uppsala, Sweden) at 20°C and 30 V for 14 h. Subsequently, IEF was conducted at 8000 V for 56 kVh for analytic analysis. Strips were then treated with equilibration buffer containing dithiothreitol (Sigma, St. Louis, MO) and then alkylated with iodoacetate (Sigma, St. Louis, MO) for 15 min. The IPG strips were transferred onto 10-18% linear gradient polyacrylamide gels (18×18 cm) and the electrophoresis was performed at 45 mA per gel at 4°C.

### Western blot

Proteins that had been resolved on 12.5% SDS-PAGE or 2-DE gels were electroblotted onto PVDF membranes using a semi-dry apparatus (GE Healthcare, Uppsala, Sweden). The membranes were blocked for 1 h with 5% non-fat milk and 0.1% Tween-20 in PBS, followed by incubation with primary antibody (3CL^pro^, NF-kB, ERK1/2, JNK, p38, GAPDH, Abcam Inc., Cambridge, MA) for 2 h at room temperature. After washing three times with PBS containing 0.05% Tween-20 (PBST), the primary antibody was detected with an anti-rabbit horseradish peroxidase-conjugated secondary antibody (Abcam Inc., Cambridge, MA) for 1 h at room temperature. Signals were developed with an enhanced chemiluminescence western blotting detection system (PerkinElmer, Boston, MA,).

## List of abbreviations

SARS-CoV: Severe acute respiratory syndrome coronavirus; 3CL^pro^: 3C-like protease; GM-CSF: human granulocyte macrophage colony stimulating factor; ELISA: Enzyme-linked immunosorbent assay; RT-PCR: reverse transcription polymerase chain reaction; IL-1β: interleukin 1 beta; IL-6: interleukin 6; IL-8: interleukin 8; TNFα: tumour necrosis factor α; IL-12p40: interleukin 12 p40 subunit; TGF-β: transforming growth factor β; ERK1/2: extracellular-signal-regulated kinases 1 and 2; NF-kB: nuclear factor kappa B; JNK: c-Jun N-terminal kinases; p38 MAPK: P38 mitogen-activated protein kinases; EGFP: enhanced green fluorescent protein; GAPDH: Glyceraldehyde 3-phosphate dehydrogenase.

## Authors' contributions

STC, GJT, and SLC participated in the design of study. MCMC, HYT, HHL, YCW, CHC, and TCH performed the experiments, collected and analyzed data. GJT, SLC, JL and MCMC wrote the manuscript. All authors read and approved the final manuscript.

## Supplementary Material

Additional file 1 **A bar graph of NF-kB and MAPKs expression in EGFP, 3CLpro, and C145A tranfected A549 cells**. The relative expression level of NF-kB and MAPKs in EGFP, 3CLpro, and C145A tranfected A549 cells at 24 and 48 hrs in Figure 6 are compared with GAPDH and depicted in the bar graph including mean and standard deviation. Click here for file
