# Supplementary figures and images for: Down-regulation of granulocyte-macrophage colony-stimulating factor by 3C-like proteinase in transfected A549 human lung carcinoma cells
Source: BMC Immunol. 2011 Feb 17;12:16. doi: 10.1186/1471-2172-12-16 (PMC3048559; doi:10.1186/1471-2172-12-16)

## Slide 1
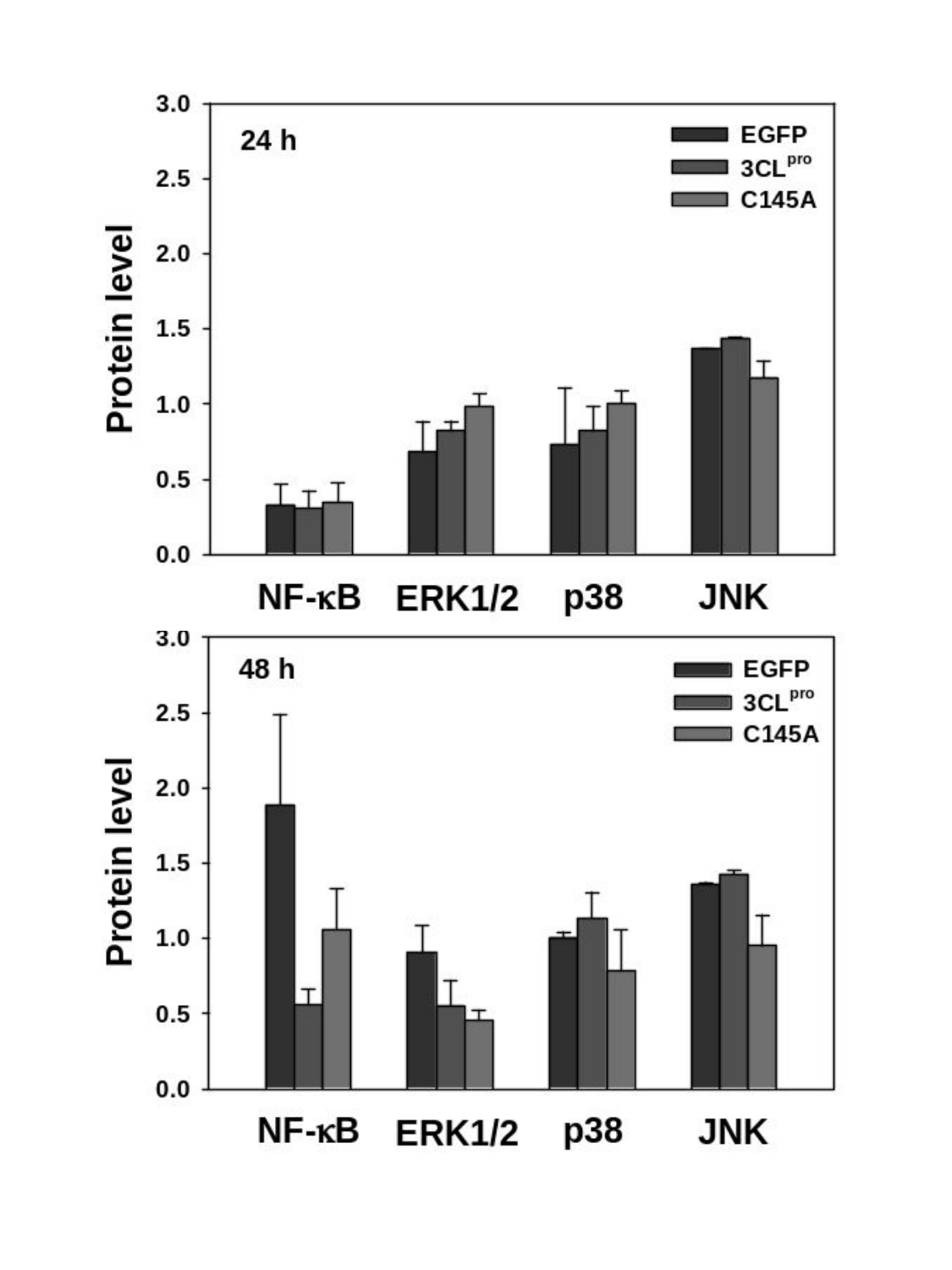

Supplement: Additional file 1 — A bar graph of NF-kB and MAPKs expression in EGFP, 3CLpro, and C145A tranfected A549 cells. The relative expression level of NF-kB and MAPKs in EGFP, 3CLpro, and C145A tranfected A549 cells at 24 and 48 hrs in Figure 6 are compared with GAPDH and depicted in the bar graph including mean and standard deviation. [file 1471-2172-12-16-S1.PPT]
